# Surgical Parameters Related to Excessive Intrarenal Pressure during Minimally Invasive Percutaneous Nephrolithotomy in the Supine Position: A Prospective Observational Clinical Study

**DOI:** 10.1155/2022/1199052

**Published:** 2022-07-12

**Authors:** Gyoohwan Jung, Yongseok Kho, Jae Suk Park, Hyeong Dong Yuk, Seung Hoon Ryang, Sung Yong Cho

**Affiliations:** ^1^Department of Urology, Seoul National University Bundang Hospital, Seongnam, Republic of Korea; ^2^Department of Urology, Seoul National University Hospital, Seoul, Republic of Korea; ^3^Department of Urology, College of Medicine, Seoul National University, Seoul, Republic of Korea

## Abstract

**Objective:**

Excessive intrarenal pressure (IRP) during surgery for renal stones is related to postoperative complications due to systemic absorption of bacteria and endotoxins. This study is aimed at evaluating factors that induce excessive IRP in minimally invasive percutaneous lithotripsy (mini-PCNL) in the supine position.

**Methods:**

27 patients underwent mini-PCNL for intrarenal stones under supine position and were analyzed in this study. The IRP changes were measured at the phases of “baseline,” “table tilting,” “upper-pole navigation,” “stone fragmentation,” and “vacuum cleaning effect.” The relationship between the mean IRP and cumulative time of IRP ≥ 30 cmH_2_O was analyzed by according to the surgical parameters. Multiple regression analysis showed the effect of the surgical parameters on postoperative fever-related IRP elevation.

**Results:**

Mean age was 59.3 ± 14.6 years. The mean stone burden was 24.6 ± 8.1 mm^3^. IRP was higher than baseline (31.6 ± 12.1) during upper-pole navigation (60.0 ± 22.9, *p* = 0.003) and stone fragmentation (46.2 ± 9.9, *p* < 0.001). The subgroup's IRP baseline < 20 cmH_2_O significantly increased during the upper-pole navigation. Changes in IRP at each stage were affected by baseline IRP (*p* < 0.001), operation methods (*p* = 0.021), number of calyces with stones (*p* = 0.034), and laser energy of Joules (*p* = 0.041) and frequency (*p* = 0.038).

**Conclusion:**

In supine mini-PCNL, the IRP was higher during laser fragmentation and upper-pole navigation. The table tilting procedure can be helpful in selected patients. The vacuum cleaner effect did not affect IRP.

## 1. Introduction

Kidney stones are a common and costly disease [1]. The surgical treatment of kidney stones is complex as there are several competing treatment modalities, and in certain cases, more than one modality may be appropriate [2]. Percutaneous nephrolithotomy (PCNL) is a standard procedure for managing large or complex kidney stones [2, 3]. However, complications, such as bleeding, collecting system injury, surrounding organ injury, infection, or sepsis, occur in approximately 15% of patients who undergo PCNL [4, 5]. Although the mechanism of infectious complication is not clearly understood, it is widely accepted that this is caused by the systemic absorption of bacteria and endotoxins via the irrigation fluid, especially under high intrarenal pressure (IRP) [6, 7].

Previous studies have demonstrated the appropriate IRP change in levels to prevent infectious complications during PCNL procedures [7–13]. However, the IRP results caused by minimally invasive percutaneous nephrolithotomy (mini-PCNL) are limited, especially in supine position. Furthermore, the analytic IRP changes as a consequence of the mini-PCNL have not yet been confirmed. The diameter of the mini-PCNL scope is smaller than that of the conventional PCNL scope, and the IRP can be different to maintain stone fragmentation. For mini-PCNL, the surgeon may perform the surgery in the supine or prone position, may tilt the table or not, may use single-tract or multiple-tract navigation, may insert a ureteral access sheath or a flexible ureteroscope only into the ureter for a combined approach, may use vacuum-cleaner effect or any suction device to evacuate the fragmented stones, or may perform stone fragmentation in the renal pelvis or the upper-pole calyx. Although surgical procedures may differ significantly, depending upon the surgeon's experience, many surgeons who want to perform supine mini-PCNL may not have the proper information on how the IRP changes as a consequence.

Therefore, we investigated the IRP change in each of the mini-PCNL procedures in the supine position without a suction device for accurate measurement. In addition, surgical parameters that cause excessive IRP during mini-PCNL procedure in the supine position were investigated, and the best surgical technique and equipment handling method were sought to effectively lower the IRP.

## 2. Materials and Methods

The study was approved by the Institutional Review Board of Seoul National University Hospital (protocol number H1901-104-1005) and registered with the clinical research information service (http://cris.nih.go.kr, KCT0007030).

### 2.1. Clinical Presentation

**Figure 1:**
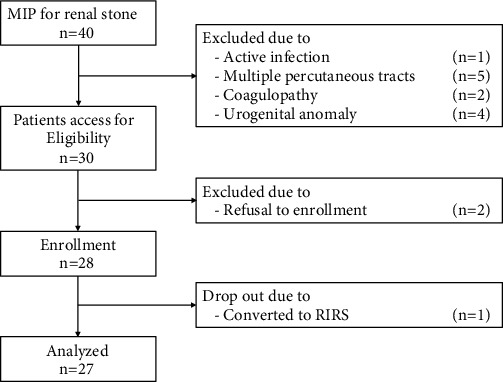
Study flow diagram.

Between July 2019 and February 2020, 28 patients, 18 years of age or older, who had undergone mini-PCNL to break intrarenal stones under supine position, were involved in this study. A single patient was dropped out, and eventually, 27 patients were analyzed. We excluded patients with active infection, multiple percutaneous tracts, coagulopathy, or urogenital anomaly, including narrow infundibulum, musculoskeletal deformities, or ureteral strictures (Figure 1). We evaluated the stones' Hounsfield units (HU), the stone size, and location. Patients underwent plain film KUB (kidney, ureter, and bladder), radiological investigation, and nonenhanced computed tomography. The stone burden was calculated as being the sum of each stone's volume. Stone free was assessed one month after surgery by postoperative nonenhanced computed tomography. We obtained informed consent from all patients for this observational study.

### 2.2. Measurement Devices of IRP

**Figure 2:**
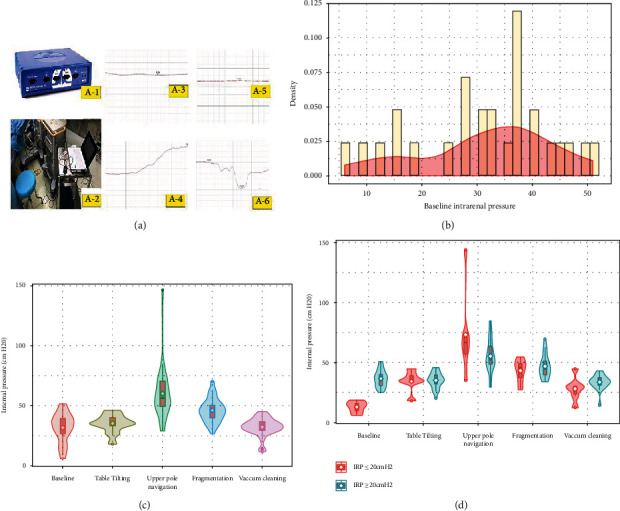
(a) Measurement of intrarenal pressure (IRP) and typical findings of pressure change according to procedures. (A-1) MP36 Student Lab system, (A-2) catheter connection, (A-3) initial IRP, (A-4) IRP during the upper-pole navigation, (A-5) IRP at the table tilting phase, and (A-6) IRP at the stone retrieval phase with the vacuum cleaning effect. (b) Initial IRP. (c) IRP change according to the procedures. (d) IRP change according to the initial IRP cut-off level at 20 cmH_2_O.

First, we connected the end of the ureteral catheter from the renal pelvis to the MP36 Student Lab system (Biopac, Goleta, CA, USA) with a baroreceptor.  Figure 2(a) shows the instrument settings that we used to measure the IRP. Next, the IRP and accumulated time were recorded simultaneously with a computer.

### 2.3. Surgical Procedures

All cases of mini PCNL were done by a single surgeon (SYC). Under general anesthesia, patients were placed in the Barts “flank-free” modified supine position [14]. We inserted a 5-Fr open-ended ureteral catheter (Cook Medical, Bloomington, IN, USA) into the renal pelvis using cystoscope and evaluated the calyceal system with contrast dye. The bladder was drained with a 12-Fr urethral Foley catheter, which was attached to the ureteral catheter. An 18-G echo-tip Chiba needle was inserted into the midpole or lower-pole calyx under ultrasonographic and fluoroscopic guidance. A 0.035-inch stiff type ZIPwire™ (Boston Scientific, Marlborough, MA, USA) was then inserted into the renal collecting system or down the ureter through the needle sheath under fluoroscopic guidance. The skin and fascia were incised, and the tract was dilated with a fascia dilator in a stepwise manner. Then, a matched metallic sheath was inserted. A size 12-Fr nephroscope (Karl Storz, Tuttlingen, Germany) was inserted through a 15/16.5-Fr metallic sheath. At the end of the procedure, we used a hemostatic agent without a nephrostomy tube.

### 2.4. Measurement of IRP In Vivo

The baroreceptor tip was positioned under fluoroscopic guidance in the middle of the renal pelvis or upper-pole calyx at the intersection of the horizontal plane of the kidney according to the surgical procedure. Then, air and liquid were flushed out of the catheter with 20 mL of normal saline in a syringe for zeroing. This process allowed stone fragments and blood clots to be washed away from the ureteral catheter or the percutaneous tract, allowing accurate pressure measurement.

We analyzed the procedures affecting IRP changes. They divided the IRP into five sections to measure it according to surgical procedures. The IRP changes were measured at the (1) “initial,” (2) “table tilting,” (3) “upper-pole navigation” of the endoscope, (4) “stone fragmentation,” and (5) “vacuum cleaning effect” phases through the percutaneous metal sheath.

1. We navigated calyces and identified the lower, middle, and upper-pole inlets. We carefully measured the initial IRP in the renal pelvis, violated it slightly to guarantee enough space to identify the target stones and perform stone fragmentation without mucosal damage. We defined the initial IRP as being reached when the surgeon could get a clear endoscopic view that allowed the surgery to be done without full dilatation of the renal collecting system following the insertion of the instrument
2. We tilted the table 10 degrees to the surgeon's side and measured the “table tilting IRP”
3. After stone fragmentation, some fragments usually stay within the upper-pole calyces during the surgical procedure because the upper-pole is usually the kidney's most dependent space in the supine position [15]. Therefore, “upper-pole navigation IRP” was measured in the inlet of the upper-pole calyx when observing the upper-pole calyx with the mininephroscope in the tilted table
4. “Stone fragmentation IRP” was measured in front of the stones during the stone fragmentation period with the holmium: YAG laser using 550 *μ*m fibers
5. “Vacuum cleaning IRP” was measured while retrieving and inserting the mininephroscope repeatedly through the percutaneous tract to remove residual fragmented stones because the fragments had been flushed out by the force of the flow and vacuum-cleaner effect from an irrigation pump (Stryker, Kalamazoo, MI, USA)

The procedures in phases 3, 4, and 5 were performed in the tilted surgical table. In order to perform ancillary retrograde intrarenal surgery (RIRS) after the main mini-PCNL procedure to remove the remnant stones [16], we inserted ureteral access sheaths into the ureter, and the pressure was measured through the ureteral catheter inside the ureteral access sheaths.

We set the laser setting to control the factors that could affect the IRP. The routine laser setting for stone fragmentation with the mininephroscope was 2.0 J with 30 Hz. In the cases of hard stones, the energy was increased to 2.5 J. To perform ancillary RIRS with flexible ureteroscopy, the laser setting was decreased to 1.0 J with 10 Hz or 20 Hz. At the end of the procedure, a 6-Fr double-J stent was inserted into the ureter using a previously placed ureteral catheter. A 12 Fr Foley catheter was inserted into the bladder.

### 2.5. Statistical Analysis

All demographic and stone parameters were reported as a mean ± standard deviation or frequency (percentage). First, we compared the IRPs of different surgical phases. Then, we analyzed the relationship between the mean IRP and cumulative time of IRP ≥ 30 cmH_2_O by multiple linear regression analysis according to the surgical parameters. Finally, we used log regression analysis to determine the effect of surgical parameters on postoperative fever-related IRP elevation. Statistical significance was defined as a *p* value < 0.05. All statistical analyses were conducted using IBM SPSS version 24.0 Software (IBM, Armonk, NY, USA).

## 3. Results

### 3.1. Demographic and Stone Characteristics of Patients

Demographics and stone parameters are presented in Table 1. The mean age of the patients was 59.3 ± 14.6 years, and 44.4% were female. The mean stone burden was 24.6 ± 8.1 mm^3^, and 22.2% of the patients had undergone other treatment for stone removal before the percutaneous procedure.

The mean initial IRP was 36.1 ± 10.8 cmH_2_0, and we found that a clear visual field could be secured during the procedure by achieving an IRP of at least 20 cmH_2_O with irrigation (Figure 2(b)).  Table 2 and Figure 2(c) show the mean IRP and its change for each phase during the procedure. The IRPs did not significantly differ between the initial and table tilting phases. However, 70.3% of the cases showed a decrease in IRP, and the mean pressure drop was 2 cmH_2_O during the phase. The IRP was higher in the upper-pole navigation phase when compared with the initial phase (60.0 ± 22.9 cm H_2_O vs. 31.6 ± 12.1 cm H_2_O, *p* = 0.003). The IRP was significantly increased during laser stone fragmentation (46.2 ± 9.9 cmH_2_O vs. 31.6 ± 12.1 cmH_2_O, *p* < 0.001). No statistically significant decrease in IRP was found when the nephroscope moved backward through the percutaneous sheath to remove the stone fragment using the “vacuum-cleaner effect.”

Figure 2(d) shows IRP changes during each phase of the procedures according to the cut-off level of 20 cm of H_2_O of the initial IRP. Patients with initial IRP ≤ 20 cmH_2_O showed significantly increased IRP during the upper-pole navigation when compared to that of patients with initial IRP > 20 cmH_2_O (69.4 ± 25.2 cmH_2_O vs. 46.7 ± 9.5 cmH_2_O, *p* = 0.006). In addition, the IRP during the upper-pole navigation in tilting also differed significantly between the two groups (8.6 ± 5.4 cmH_2_O vs. 35.6 ± 25.1 cmH_2_O, *p* = 0.001). The IRP dropped during stone fragmentation (45.5 ± 8.3 cmH2O vs.46.6 ± 10.7 cmH2O, *p* = 0.781) following upper-pole navigation. However, there was no significant difference in IRP between the two groups during stone fragmentation.

### 3.2. Correlation between IRP, the Stone Characteristics, and the Elements of Surgical Setting

Table 2 shows that the stone characteristics and the surgical setting elements were related to IRP. In multiple regression analysis, the mean IRP > 20 cmH_2_O was related to the number of calyces with stones, high-laser energy (2 J with 30 Hz), and initial IRP value. In univariate logistic regression analysis, higher mean IRP was related to initial IRP ≥ 30 cmH_2_O (OR 7.333, 95% CI 1.163-46.235, and *p* = 0.034), but it was not significant in multivariate analysis (Table 3). Only age ≥ 60 was significantly related to higher initial IRP ≥ 30 cmH_2_O (OR 199.852, 95% CI 2.387-16733.033, and *p* = 0.019) (Table 4).

## 4. Discussion

This study is aimed at analyzing the effects of IRP settings during mini-PCNL in supine position. Although previous studies have analyzed IRP according to the size of the PCNL tract [17–19], for the first time to the best of our knowledge, this study tries to analyze the IRP changes during supine mini-PCNL according to operative technique to lower pyelovenous backflow and infectious complications caused by excessive IRP.

In our study, the IRP was changed according to different phases of the supine mini-PCNL procedures. The IRP peaked during upper-pole navigation across all procedures. In the supine position, the upper-pole is usually the most dependent space of the kidney. Following stone fragmentation, the fragments typically stay within the upper-pole calyces during the latter part of the surgical procedures. Therefore, the navigation time to remove all the fragments can be prolonged. An interesting finding is that the IRP changes in upper-pole navigation were found to be greater in the group with an initial IRP ≤ 20 cmH_2_O than in those with an initial IRP > 20 cmH_2_O. This can be hypothesized that the kidneys in which the IRP was kept relatively low would be more sensitive to IRP changes than those in which the IRP was kept high [9]. Therefore, we should shorten the time for upper-pole navigation as far as possible. The cumulative time for upper-pole navigation should be minimized as much as possible, especially when removing the fragments. In this study, the IRP was higher than 40 cmH_2_O in some cases because of peak pressures during the upper-pole navigation procedure. However, the increase of IRP above 30 cm _2_O did not last for >60 seconds. A febrile episode during the postoperative period occurred in two cases, and it was difficult for us to find the relationship between the IRP and the febrile episodes.

The higher IRP during upper-pole navigation and more changes in the lower baseline IRP group are important because this may be related to complications after mini-PCNL [20–22]. Although animal experiment has limitations, Wang et al. have found that renal tubules show histologic changes under prolonged exposure to pressure > 20 cmH_2_O [23]. In a canine study by Hinman, pyelovenous backflow occurred at renal pressures > 30 to 35 cmH_2_O [22]. Other clinical trials also recommend maintaining the IRP < 30 cmH_2_O to prevent pyelovenous backflow [5]. These results are consistent with other clinical studies that have investigated infectious complications following PCNL. Previous studies have shown that the factors associated with postoperative fever and infectious complications following PCNL include an average IRP higher than 20 cmH_2_O during the whole procedure and an accumulated time with IRP ≥ 30 mmHg longer than 50-60 seconds [7, 12, 13]. Zhong et al. compared the IRP differences and infectious complication frequencies according to the PCNL tract size [13]. These results show that fever and infectious complications frequently occur when the mean IRP is >20 cmH_2_O during the procedure or when there is a cumulative time of >50 seconds at which the IRP is kept at >30 cmH_2_O. Similarly, Wu et al. found that a mean IRP > 20 cmH_2_O at >60 seconds of cumulative time or IRP > 30 cmH_2_O were significantly related to postoperative fever [12]. From these results, we set a cut-off value that can cause pyelovenous backflow and infectious complications for IRP at 20 cmH_2_O. Our study did not demonstrate a statistical association between high IRP and postoperative infection, which may be attributed to the small number of cases and events. It is considered necessary to prove this through larger studies.

In this study, table tilting on the surgeon's side did not show any significant IRP changes. Although 19 cases (70.4%) showed a pressure drop, the decrease ranged 1 to 5 cmH_2_O. This may be because of rotational movement of the kidney and the resultant descent of the tip of the percutaneous tract. However, eight (29.6%) cases showed an IRP increase following table tilting, and three of them showed an IRP increase at around 20 cmH_2_O. These three cases showed hypermobility of the kidneys during the percutaneous puncture, and we may hypothesize that kidney hypermobility cannot decrease the IRP right after percutaneous puncture because the kidneys would be positioned relatively more immediately than the kidneys without hypermobility.

We thought that the IRP might increase during the stone fragmentation because laser emissions can make the stone undergo retropulsion, transmitting energy through the irrigation fluid. However, no significant IRP increase was found. Additionally, the IRP can theoretically decrease because of the “vacuum cleaner effect” during the stone retrieval procedure via the percutaneous tract, creating negative pressure between the nephroscopic tip and the targeted stones. Our results, however, showed that the IRPs during the laser emission procedures and the vacuum cleaner effects did not differ because the laser emission and vacuum cleaner effects had only a local effect.

Recently, Gökce et al. [24] reported that active aspiration significantly lowers intrapelvic pressure and keeps it <40 cmH_2_O. During the study, the pressure was measured in the mini-PCNL under supine position for 90 seconds in 4 different settings with respect to the location of the tip of the sheath and nephroscope. However, the pressure was not measured for each surgical procedure. Our study measured IRP associated with surgical procedures in a controlled setting, which provides a more comprehensive aspect of changes in pressure that occur in various situations during surgery.

The initial IRP seems to be the most critical factor influencing the mean IRP. There may be several reasons for the high initial IRP, presumably related to such as decrease in urine drainage due to ureteropelvic junction obstruction or ureteral stricture, decrease in compliance of renal collecting system, and individual variation in kidney location or anatomy. According to multivariate logistic regression analysis, age over 60 years was the only factor that was related to high initial IRP in our data. Fibrotic change of renal collecting system or ureter which is commonly accompanied in older patients might be one possible explanation of this result. Other comorbidities may have affected, but the analysis could not be conducted due to lack of data. Further research on this subject is expected.

The current study has following limitations. First, measuring pyelovenous backflow by inserting a pressure meter in retrograde fashion through the ureter may have a technical pressure transmission error. Second, this study mainly targeted complex renal stones, and the average pressure may have been measured higher than the usual procedures because of the limited intracaliceal space. However, despite these limitations, this study analyzed IRP changes according to operative technique and the various technical factors that may affect the pyelovenous backflow and the occurrence of infectious complications during the supine mini-PCNL procedures.

In conclusion, during supine mini-PCNL, an increase in IRP and the cumulative surgical time for the upper-pole navigation should be minimized, and the pressure-lowering technique is necessary. The table tilting procedure can be helpful in selected patients. Stone fragmentation by laser emission and the vacuum cleaner effect did not significantly change the IRP.

## Figures and Tables

**Table 1:** Demographics and stone parameters.

Variables		*p* value
Patient number (*N*)	27	
Mean age (years)	59.3 ± 14.6	
Gender (male:female)	15 (55.6%) : 12 (44.4%)	
Mean BMI (kg/m^2^)	24.7 ± 4.2	
Comorbidities		
Diabetes mellitus	7 (29.2%)	
Hypertension	8 (33.3%)	
Cardiovascular disease	2 (7.4%)	
Pulmonary disease	2 (7.4%)	
Stone laterality (right:left)	15 (55.6%) : 12 (44.4%)	
Stone burden (mm^3^)	24.6 ± 8.1	
Stone number	2.6 ± 1.7	
Hounsfield unit	1286.8 ± 567.3	
Stone location		
Pelvis	16 (59.3%)	
Upper calyx	4 (14.8%)	
Mid calyx	6 (22.2%)	
Lower calyx	17 (63.0%)	
Multiple	13 (48.1%)	
Previous stone treatment history	6 (22.2%)	
Staghorn stone	12 (44.4%)	
Hydronephrosis	11 (40.7%)	
Complete stone free rate	23 (85.1%)	
Clinically stone free rate (<2 mm)	25 (92.6%)	
Postoperative infection	2 (7.4%)	
IRP during different phases of procedures		
Initial	31.6 ± 12.1 (6-51)	Reference
Table tilting	35.1 ± 7.2 (18-46)	0.376
Upper-pole navigation	60.0 ± 22.9 (18-46)	0.003^∗^
Stone fragmentation	46.2 ± 9.9 (29-145)	<0.001^∗^
Vacuum cleaning effect	32.3 ± 7.6 (12-45)	0.967

^∗^ *p* < 0.05. BMI: body mass index; IRP: intrarenal pressure.

**Table 2:** The multiple regression analysis of surgical parameters related to mean IRP > 20 cmH_2_O.

	Beta	SE	std. beta	*t* value	*p* value
Stone burden, maximal diameter (mm)	-0.01	0.01	-0.31	-1.575	0.128
Hounsfield unit	0	0	0.24	1.229	0.231
Number of calyces with stones	0.15	0.07	0.42	2.252	0.034^∗^
Number of stones	0.01	0.01	0.12	0.604	0.552
Hydronephrosis	0.18	0.16	0.22	1.107	0.279
Previous cortical defect	-0.18	0.19	-0.2	-0.979	0.337
Number of percutaneous tract	-0.26	0.2	-0.26	-1.304	0.205
Laser energy setting (J)	-0.73	0.34	-0.4	-2.163	0.041^∗^
Laser energy setting (Hz)	0.05	0.02	0.41	2.201	0.038^∗^
Laser using time (min)	0	0	-0.13	-0.656	0.518
Initial intrarenal pressure (cmH_2_O)	0.03	0	0.83	7.192	<0.001^∗^
*R* − square = 0.686					

^∗^ *p* <0.05.

**Table 3:** The logistic regression analysis of surgical parameters related to mean IRP ≥ 30 cmH_2_O.

	Univariate			Multivariate		
Variables	OR	95% CI	*p* value	OR	95% CI	*p* value
Stone burden, maximal diameter (mm) ≥ 20	1.100	0.179-6.755	0.918	0.962	0.060-15.432	0.978
Hounsfield unit ≥ 1000	0.593	0.122-2.887	0.517	0.482	0.062-3.755	0.486
Number of stones ≥ 3	1.143	0.250-5.224	0.863	0.972	0.091-10.381	0.981
Hydronephrosis	1.543	0.329-7.226	0.582	0.964	0.142-6.560	0.970
Previous cortical defect	1.100	0.179-6.755	0.918	0.900	0.078-10.359	0.933
Number of percutaneous tract ≥ 2	0.667	0.093-4.803	0.687	0.713	0.009-58.840	0.881
Laser energy setting (J) 1.5 J	ref.			ref.		
2.0 J	1.000	0.055-18.085	1.000	1.916	0.006-624.781	0.826
2.5 J	0.500	0.013-19.562	0.711	1.601	0.000-6040.09	0.911
Laser using time (min) ≥ 30	1.111	0.213-5.802	0.901	1.322	0.067-26.037	0.854
Initial IRP (cmH_2_O) ≥ 30	7.333	1.163-46.235	0.034	8.005	0.842-76.110	0.070

**Table 4:** The multivariate logistic regression analysis related to initial IRP ≥ 30 cmH_2_O.

Variables	OR	95% CI	*p* value
Age (yrs) ≥ 60	199.852	2.387-16733.033	0.019
Body mass index (kg/m^2^) ≥ 23	22.810	0.409-1271.522	0.127
Stone burden, maximal diameter (mm) ≥ 20	0.082	0.001-7.820	0.283
Hounsfield unit ≥ 1000	18.762	0.447-787.017	0.124
Hydronephrosis	18.746	0.531-661.629	0.107
Previous cortical defect	1.215	0.058-25.680	0.900
Number of stones ≥ 3	0.369	0.018-7.702	0.520

## Data Availability

The data used to support the findings of this study are available from the corresponding author upon request.
